# Prior expectations about where other people are likely to direct their attention systematically influence gaze perception

**DOI:** 10.1167/16.3.7

**Published:** 2016-02-05

**Authors:** Peter C. Pantelis, Daniel P. Kennedy

**Affiliations:** pcpantel@indiana.edu www.indiana.edu/~brainlab; dpk@indiana.edu www.indiana.edu/~brainlab; Department of Psychological and Brain Sciences, Indiana University-Bloomington, Bloomington, IN, USA

**Keywords:** *eye gaze perception*, *Bayesian modeling*, *social perception*, *visual attention*, *salience*

## Abstract

Different locations in the visual environment vary greatly in terms of how likely they are to draw a person's attention. When inferring the most likely target of another person's gaze, it would therefore be a reasonable strategy to incorporate expectations about the relative visual salience of these various locations, weighing this prior knowledge against incoming social signals (e.g., eye cues). This Bayesian approach to modeling gaze perception has informed computer vision techniques, but whether this model accounts well for human performance remains an untested hypothesis. We present subjects with a “gazer” fixating his eyes on various locations on a two-dimensional surface, and project arbitrary photographic images onto that surface. Subjects judge where the gazer is looking in each image. A full Bayesian model, which takes image salience information into account, fits subjects' gaze judgments better than a reduced model that only considers the perceived direction of the gazer's eyes. Varying the amount of time the subject is allowed to view the gazer reveals that center bias tends to dominate gaze judgments early, whereas salient features specific to the projected image influence judgments at longer viewing durations.

## Introduction

The target of another person's gaze is a strong cue for where that person is directing his or her visual attention, and therefore what may be on his or her mind moment to moment (Pärnamets, Johoansson, Hall, Balkenius, & Spivey, 2015). Additionally, because people (and other animals) tend to direct their visual attention to the informative and behaviorally relevant areas of the environment (Mackworth & Morandi, 1967), the ability to infer another's attention (via gaze, as a proxy) also helps to reveal the important things that may be happening in a person's immediate vicinity (Byrne & Whiten, 1991).

The direction of another person's eye fixation is a robust and precise cue for tracking gaze (and therefore, attention), and it is therefore unsurprising that the human visual system has evolved to process this social signal with remarkable accuracy and efficiency (Cline, 1967; Gale & Monk, 2000; Symons, Lee, Cedrone, & Nishimura, 2004; Bock, Dicke, & Thier, 2008). However, no perceptual signal is perfectly noiseless in its extraction and unambiguous in its interpretation. As such, secondary cues like head position (Wallaston, 1824; Ken, 1990; Langton, 2000) or even facial expression (Martin & Rovira, 1982; Lobmaier, Tiddeman, & Perrett, 2008) concurrently inform the judgment of where another person is looking.

But additionally, if one had reliable intuitions about where in the visual scene another person would be likely to direct his or her gaze—a priori of extracting the signal from his or her eyes—then this contextual information could potentially be integrated with the eye cue to improve the inference of gaze direction. Past experiments have indeed demonstrated the influence of context on human gaze perception, with people showing a bias that another person's gaze is directed toward them (Ken, 1990; Mareschal, Calder, & Clifford, 2013) or at objects (Lobmaier, Fischer, & Schwaninger, 2006; Wiese, Zwickel, & Müller, 2013). Each of these individual empirical findings make sense given basic intuitions about human nature—that is, objects and faces would naturally be regions of interest in a counterpart's visual scene (Yarbus, 1967), and even the most mundane face is surely more interesting than, say, the empty space immediately to the left and right of it.

But in turn, it should be clear that all of the locations in the counterpart's visual environment (including one's own face) are salient to varying degrees—that is, a priori more or less likely to capture the other person's visual attention. We appeal to the more general case, and predict that prior considerations with respect to presumed visual salience should systematically factor into human gaze perception. This basic approach—combining perceptual cues from the target person's eyes (or head position, etc.) with the visual salience of the scene—has been exploited to improve the accuracy of computer vision algorithms in both the discrimination of gaze direction (Hoffman, Grimes, Shon, & Rao, 2006; Yücel et al., 2013) and in the related task of identifying where another person is pointing (Schauerte, Richarz, & Fink, 2010). We here test whether human gaze perception employs a similar mechanism, asking whether the performance of a model like this would be consistent with an observer's judgments of the most likely target of another individual's gaze (regardless of whether the observer's judgment is correct with respect to ground truth).

Our experimental subjects view photographs of a young man gazing at various locations on a partially transparent surface situated between him and the camera. The experimental task is to indicate where on this surface this “gazer” is looking, a task that we defined computationally as the inference of the location [*x,y*] within the continuous two-dimensional (2-D) plane where the photographed individual is gazing within the continuous two-dimensional (2-D) plane (*G_x_*_,_*_y_*) given the gaze directional cue from the eyes of the person (*D*) and the image presented in that plane (*I*). Bayes' rule yields the posterior probability distribution, continuous over the 2-D hypothesis space:

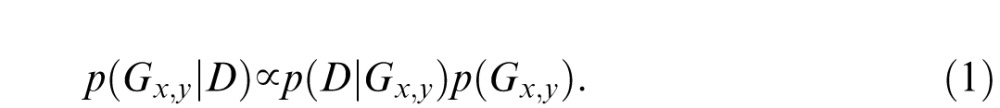



In our treatment, the prior—*p*(*G_x_*_,_*_y_*)—is equivalent to the relative visual salience of location [*x*, *y*] within image *I*, where salience is some model of where people are a priori likely to direct their visual attention and fixation. This study explores whether a Bayesian model that incorporates a visual salience map as a prior can account for actual human subjects' gaze judgments better than a model that ignores this information, and uses only the eye cues.

## Experiment 1

In Experiment 1, we presented subjects with a gazer fixating his eyes on various locations on a 2-D surface, and projected arbitrary photographs onto that surface. We developed two models—a full Bayesian model that takes the relative a priori salience of locations in the image into account, and a reduced model that only considers the perceived direction of the gazer's eyes—and assessed how well these models predicted subjects' judgments of where the gazer was looking.

### Methods

#### Subjects

All subjects gave written informed consent in accordance with the tenets of the Declaration of Helsinki. Twenty-three undergraduates at Indiana University received course credit for their participation in the experiment.

#### Stimuli: Photographs of the gazer

We took a set of photographs of a young man (the “gazer”) seated behind a glass surface. In each photograph, the gazer fixated his eyes on a different location on the glass surface, where a grid of points had been marked (later, these marks were digitally removed from the photographs, leaving no observable trace). Though other cues (such as head position) can also be exploited to infer the target of gaze, for this experiment we aimed only to vary the eye cues among these photographs. Therefore, the gazer maintained minimal head and body movement as he fixated on the various locations on the glass surface.

The height of the origin of this grid of points, the camera lens, and the center point between the gazer's eyes was 125 cm. The glass surface was 115 cm from the gazer's face, and 160 cm from the camera. The gazer's face was lit from above, both from the left and right, so as to avoid casting heavy shadows on his face. The photographs were taken with a Canon EOS Digital Rebel XT camera, a 50-mm lens, 1/125-s exposure time, and no flash. The original resolution of these photographs was 3456 × 2304 pixels.

Thirty-three photographs were used in the experiment. One of these photographs was taken with the gazer fixating on the origin (i.e., straight ahead, and directly into the camera), and the other 32 photographs were taken with the gazer fixating on 32 respective marks arranged in a lattice of seven rows and nine columns. The first, third, fifth, and seventh rows of this lattice each consisted of five marks, evenly spaced at 10-cm intervals. The second, fourth, and sixth rows of this lattice each consisted of four marks, also evenly spaced at 10-cm intervals, but offset by 5 cm with respect to the odd rows.

The experiment was presented on a 2560 × 1440 pixel display. One of the 33 photographs of the gazer appeared in every trial of the experiment, within a 1200 × 800 pixel window at the center of the display. The unused, background portion of the display (falling outside of the edges of the 1200 × 800 pixel window) was made gray.

**Figure 1:**
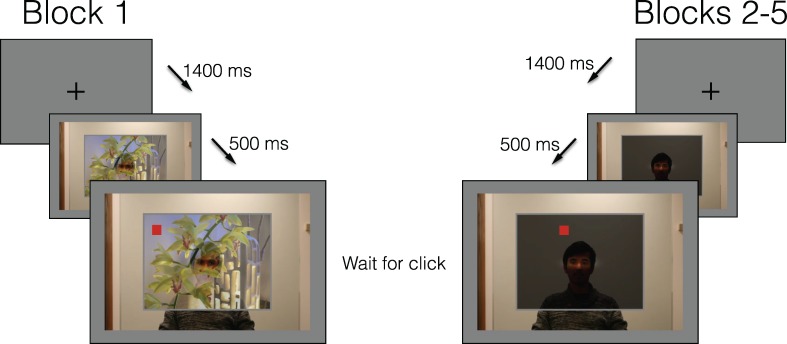
Experiment 1. After the presentation of a fixation cross for 1400 ms, the scene appeared. After 500 ms, a mouse cursor appeared as a red square at a random location within the projected image (this image was a photograph in Block 1, and uniform gray in Blocks 2–5). The subject indicated with a mouse click where he or she thought the gazer was looking. After the subject clicked, the next trial began. (Note: The fixation crosses and red mouse cursors are enlarged in this Figure to be more visible.)

For every trial, a rectangular gray frame (inner dimensions: 550 × 733 pixels; outer dimensions: 570 × 753 pixels) was superimposed on the photograph. When the gazer had been photographed, he had always fixated on locations that would have fallen within this gray frame. Either an image (for Block 1) or uniform gray (for Blocks 2–5) was presented within the rectangular gray frame in each presented scene, and alpha blended (at *α* = 180, where 0 is fully transparent and 255 is fully opaque) with the background photograph of the gazer (see Figure 1). For the subject, this created a perceptual effect akin to the subject and gazer being on opposite sides of a partially transparent surface, with the gazer's silhouette faintly visible through it. Only a tight ellipse around the gazer's eyes was fully visible through the image, with the area around the eyes smoothly transitioning to greater opacity. Thus, in either condition (projected image, or uniform gray), the gazer's eyes were made fully visible to the subject, and presented simultaneously with the supposed target of his gaze.

#### Stimuli: Projected images

For the first block of trials, images were projected onto the plane upon which the gazer had fixated. The 165 color images (a subset of a pool of images provided by Judd, Ehinger, Durand, & Torralba, 2009) included a wide range of indoor and outdoor scenes, 51 of which contained people. We selected this subset of 165 images from the larger pool on the basis that they were all of a consistent size (768 × 1024 pixels). For this experiment, these images were resized to fit the presented 550 × 733 frame.

#### Procedure

The experiment was programmed in MATLAB using the Psychophysics Toolbox (Brainard, 1997; Pelli, 1997). It consisted of five blocks, each consisting of 165 trials. The subject took a 5-min break after the third block.

Before the first trial of each block, four photographs were displayed in succession, each for 1 s. In these four photographs, the gazer was fixated on four respective locations (marked with 8- × 8-pixel black squares) near the four respective corners of the gazed-upon glass surface. This was a calibration of sorts for the subject, who could get a sense of how the gazer's eyes were positioned when he had been photographed fixating on the extremes of the glass surface.

Each trial began with a black fixation cross, presented at the center of the screen for 1.4 s against a gray background. The subject was then presented with a static scene. Over the course of each block of scenes, each of the 33 photographs of the gazer (fixated on 33 respective locations) was featured five times, with these 165 total trials being randomly shuffled.

For the first block, one of 165 color images (from the Judd et al., 2009 set) was randomly assigned to each of these 165 trials and projected into the frame in front of the gazer; thus, the projected images and the photographs of the gazer were randomly paired, and the contents of the respective images varied independently of the actual target of gaze. Though the scenes were perceptually realistic, the subject was not explicitly instructed that the gazer was (or was not) truly gazing upon an actual physical image present in front of him when the photographs had been taken. Upon debriefing, most subjects expressed skepticism that the gazer was actually looking at the photographs, especially after having viewed multiple trials in which the gazer was seemingly fixating on irrelevant areas of the images.

For the second through fifth blocks, the frame in front of the gazer was filled with a uniform gray. Five hundred milliseconds after the presentation of this scene, a 10- × 10-pixel red square appeared at a random location within the frame, and could be controlled with the mouse. After the time when this red cursor appeared, the subject could indicate with a mouse click where, within the frame, he or she believed that the gazer was looking. There was no enforced time limit for this task, and the entire scene remained on the screen until the subject responded. After the subject clicked, the next trial began. The experimental procedure for each trial is illustrated in Figure 1.

### Bayesian model

#### The likelihood: Using cues from the eyes of the gazer

Computational treatments of the problem of discriminating the target of another person's gaze from eye and head cues (e.g., Kim & Ramakrishna, 1999; Hoffman et al., 2006; Yücel et al., 2013; Gao, Harari, Tenenbaum, & Ullman, 2014) often model gaze as a vector or blurry cone emanating from the gazer's face and intersecting with surfaces in the environment. A complete, self-contained algorithm for judging another person's gaze would employ one of these rigorous computer vision approaches in order to compute what we here define as the likelihood function: *L*(*G_x_*_,_*_y_* | *D*).

We instead derive the likelihood function empirically from each subject's gaze judgments recorded during Blocks 2–5. (These were the trials for which the gazer was presented as viewing uniform gray surface.) We associate each photograph of the gazer—associated with the gazer's eyes being fixated in one of 33 directions—with a 2-D likelihood function, which we assume to be elliptical (a bivariate Gaussian distribution). This assumption of an elliptical shape makes sense if one imagines a cone of gaze emanating from the gazer's eyes (see also Gamer & Heiko, 2007), because the intersection of this cone with the gazed-upon planar surface would be elliptical in shape (indeed, this is the geometric definition of an ellipse, one of the basic types of conic section).

**Figure 2:**
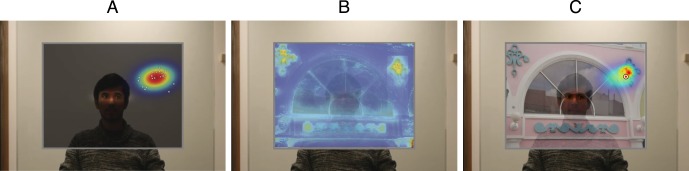
Left: Likelihood. Subjects indicated where they thought the person in the photo was looking, within a uniform gray area. The “gazer” was shown fixating on each of 33 target locations within the frame, 20 times per subject. Here, the white dots represent the 20 locations selected by one actual subject (via mouse click) when presented with this same scene. We fit a Gaussian ellipse to these 20 points (superimposed here on the scene), and this ellipse enters into the computational model as the likelihood function with respect to this particular directional cue from the eyes of the gazer. Center: Prior. During the first block of the experiment, images were projected into the frame, and subjects indicated where in the picture they thought the person in the photo was looking. Here, we superimpose the salience map corresponding to this particular image, a continuous 2-D function that enters into the computational model as the prior. Right: Posterior. The posterior probability outputted by the Bayesian model (superimposed here on a screenshot from the experiment) is a multiplication of the likelihood function (given this gaze direction) and prior (given this image). For this particular trial, we present one possible location a subject may have clicked, as a small white bullseye. We assess the model's performance on a given trial as the likelihood of the subject's gaze judgment given the model's posterior prediction map.

After collecting responses from each subject as he or she cycled 20 times through the complete set of 33 eye directions, we estimated the mean (*μ*) and 2 × 2 covariance matrix (Σ) of all 33 Gaussian ellipses comprising a complete set of personalized likelihood functions. Each of these probabilistic 2-D likelihood maps was renormalized to sum to 1. For an example of one elliptical likelihood map derived for one experimental subject with respect to one of 33 directional cues from the gazer's eyes, see Figure 2A.

#### The prior: Using contextual salience information

We hypothesized that it would be expedient for the human visual system to exploit context in the service of a predictive model of where other people are a priori likely to look in a scene. Many computer vision models have already been developed to serve precisely this function—predicting where human observers are likely to fixate their visual attention in a given image (e.g., Itti, Koch, & Niebur, 1998; Harel, Koch, & Perona, 2006; Tavakoli, Rhatu, & Heikkilä, 2011)—and the performance of many of these models has been systematically benchmarked (at saliency.mit.edu).

The algorithm put forth by Judd et al. (2009) incorporates low-level visual features (e.g., intensity and color contrast), higher level features (e.g., face detection), and a prior bias toward the center. We use their salience model because they make freely available (a) MATLAB code for their salience model, (b) a set of images against which their salience algorithm has been validated and against which other algorithms have been tested for comparison, and (c) precomputed salience maps corresponding to these images. In our Bayesian treatment, we set the prior—corresponding to the gazed-upon image in the scene—to a 2-D map of the relative visual salience of locations within it (as defined by the Judd et al. algorithm). This computed salience serves as a simplified proxy (i.e., a model) for the subject's hypothesized expectation of which locations in a scene would be more or less likely to draw the gazer's visual attention.

**Figure 3:**
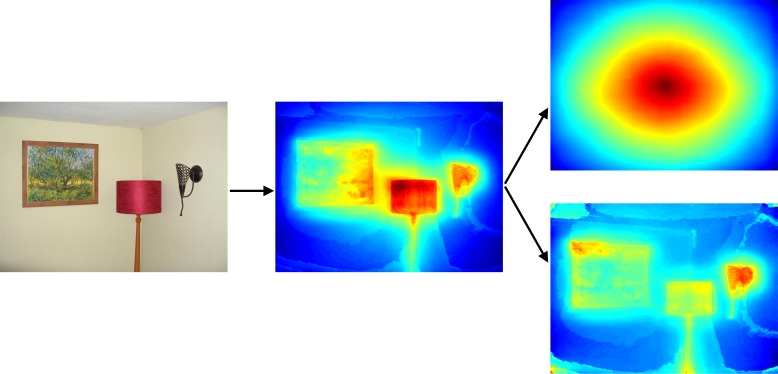
Left: One of the images from the stimulus set. Center: The corresponding salience map, as computed via the Judd et al. (2009) algorithm. Right: The salience map for this image is divided by the average salience map (top) to yield the normalized salience map employed by the Bayesian model (bottom).

We made one further adjustment to the Judd et al. (2009) salience maps before they entered into the computational model. As explained in the previous section, our Bayesian model of human gaze perception employs a likelihood function that is derived empirically from judgments the individual subject makes about where the gazer is looking within a uniform gray surface. Thus, the subject's spatial biases (namely, center bias) will already be largely accounted for via the likelihood. However, the centers of the Judd et al. salience maps tend to be more salient because (a) a strong, explicit center bias is a feature of the Judd et al. algorithm, and (b) high- and low-level features tend, empirically, to appear toward the centers of images. Thus, using these salience maps without first correcting for this center bias will result in a computational model that double counts this global tendency. To create salience maps that better reflect local features of individual images, we first calculated the average salience map across the set of 165 maps that corresponding to the images in our set. We then divided each of the 165 salience maps by the average salience map, resulting in a set of maps for which no spatial location was systematically more salient than any other location across the set. We incorporate these adjusted salience maps as the prior in our Bayesian model of human gaze perception (see Figure 2B for an example of a salience map corresponding to one of the 165 images in our stimulus set; see Figure 3 for an illustration of how we derived each map.)

#### The posterior: Combining the eye cue with image salience

Each scene observed by the subject during the first block of the experiment featured a photograph of the gazer fixating in one particular location on a 2-D surface, and one arbitrary image projected onto that gazed-upon surface. With respect to each scene, the posterior prediction of the Bayesian model is the pixel-by-pixel multiplication of the likelihood function (associated with the individual subject viewing the gazer fixating in one particular direction; *p*[*D* | *G_x_*_,_*_y_*]) and prior (i.e., the probabilistic salience map computed for the image; *p*[*G_x_*_,_*_y_*]). After this multiplication, the posterior distribution is renormalized to sum to 1. The resulting prediction is a hybrid of the two maps giving rise to it, exploiting the local salience within the neighborhood of locations where the gazer may have plausibly been looking, given the direction of his eyes. See Figure 2 for an illustration of how the likelihood and salience prior are combined to yield the posterior distribution outputted by the full Bayesian model.

### Results

#### Validation of the likelihood model

Each subject's personalized likelihood function—a set of 33 ellipses fit to his or her 660 gaze judgments made during Blocks 2–5—was first assessed and optimized via a cross-validation procedure. A set of ellipses was fit to the subject's responses during three of these blocks of trials, and tested on how well it predicted responses on the fourth block. This leave-one-out cross-validation was performed each of the four possible ways (leaving each of the four blocks out as the test set).

After fitting a set of 33 Gaussian ellipses to a training set of three blocks, the main diagonals of their covariance matrices Σ were multiplied by one additional parameter, which was optimized for each subject via this same cross-validation procedure. Increasing the variances of these ellipses in this manner prevented overfitting to the training set and improved the model's ability to predict the subject's gaze judgments during the remaining test block. For most subjects, multiplying the variances of the fit ellipses by 1.6 proved to be optimal.

The cross-validated performance of the likelihood model was good and remarkably consistent across subjects. For only one very atypical subject, we were unable to validate the likelihood model—that is, no parameterization of the likelihood model trained on any three of the subject's blocks was able to predict the subject's gaze judgments on the remaining test block above chance. We therefore excluded this subject from subsequent analyses.

#### Model assessment and comparison

We evaluated the full Bayesian model in direct comparison with a model that only relied on the perceptual signal from the eye cues of the gazer (i.e., the unadorned likelihood model, not multiplied with the salience map). We tested the relative performance of these two models in predicting the gaze judgments made by subjects during the first block of the experiment. During these critical trials, the subject viewed scenes in which the gazer was presented with a projected image—unlike in Blocks 2–5, in which the gazer was presented with a uniform gray surface.

Because the likelihood function (a component of both the full Bayesian model and the reduced model) was independently validated and optimized for each subject with respect to data collected during subsequent blocks, neither the full Bayesian model nor the reduced model fit any free parameters to judgments made by the subjects during the critical first block. Therefore, although the full Bayesian model is more computationally elaborate, the relative performance of the two models can be assessed on equal footing without making a correction for model complexity (for example, with Akaike information criterion; Burnham & Anderson, 2004).

The relative performance of these two models was first assessed in terms of log likelihood ratio. For a given trial, the gaze judgment made by the subject had a likelihood given the prediction maps of either model (e.g., as in Figure 2A, C). Over each subject's 165 trials, the predictions of the two models were compared via their cumulative likelihood ratio. The natural logarithm of this ratio was computed for each subject, with positive values favoring the full Bayesian model and negative values favoring the reduced “eye cues only” model. By this measure, the cumulative log likelihood ratio across all subjects (101.9) very strongly favored the full model.

To estimate the extent to which individual subjects used the salience prior (and to rule out the possibility that the previous strong result was driven by only a few outlier subjects), we fit a parameter (*δ*) to each subject's data, optimizing the full Bayesian model with respect to the likelihood of the subject's judgments under the various possible settings of *δ*:

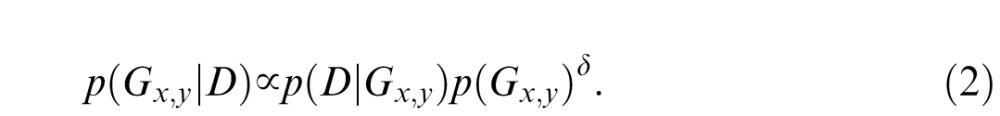



**Figure 4:**
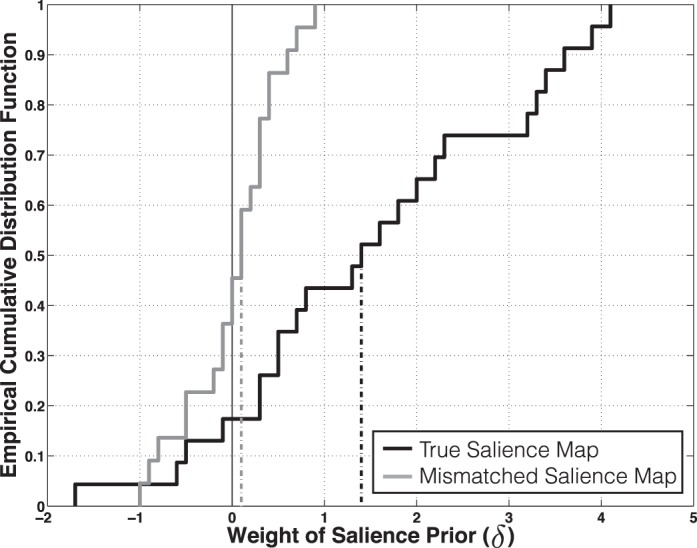
Each of the 22 subjects is represented as an increment in these empirical cumulative distribution functions. Using a salience map as a prior improves the performance of the model with respect to the judgments of 18 out of 22 subjects (black; median *δ* = 1.4). By comparison, using a mismatched salience map as a prior does not systematically improve performance (gray; median *δ* = 0.1).

If *δ* were set to 0 for an individual subject's best-fitting model, then the addition of the salience map did not systematically improve (or hurt) the performance of the model. The higher the *δ*, the more weight the subject apparently assigned to the salience cue (Figure 4).

In this case, the revised Bayesian model estimated an additional parameter from each subject's Block 1 data; we therefore rely on a parametric *t* test to assess the success of this model compared to chance. The mean subject optimally weighted the salience cue at *δ* = 1.5, significantly above zero, *t*(21) = 4.48, *p* < 0.001.

These data confirmed our hypothesis that subjects would exploit prior information about the relative salience of locations in the gazed-upon image, in addition to using the directional cue from the gazer's eyes. To provide additional context for assessment of our model, we reran the full Bayesian model, but instead of feeding the model the appropriate salience map corresponding to the gazed-upon image in a given trial, we mismatched each image with a salience map corresponding to one of the other 164 images in the set. The motivation for the assessment of this mismatched Bayesian model was to examine whether the true Bayesian model had improved the performance of the reduced “eye cues only” model for some superficial reason that was not specific to features of the particular image.

Whereas using the true salience maps had consistently improved the performance of the reduced “eye cues only” model across subjects, using mismatched salience maps only made the performance of the model worse, such that one would have been far better off using the reduced model (cumulative log likelihood ratio = −57.9). Repeating the parameter-fitting procedure used to estimate the extent to which each subject used the salience prior, we found that the optimal model for each subject, on average, did not assign any weight to this mismatched salience map (mean *δ* = 0.0; *t*[21] = 0.13, *p* = 0.90). Thus, incorporating a mismatched salience map merely added noise to the model—not just any prior will do.

## Experiment 2

In Experiment 2, we again presented subjects with scenes featuring the gazer fixated on various locations on a semitransparent surface. In all trials of Experiment 2, an arbitrary image was projected onto that surface (as in Block 1 of Experiment 1). To examine the time course of the salience effects observed in Experiment 1, as well as the influence of spatial biases at different viewing durations, we manipulated the amount of time subjects were allowed to view these scenes before judging the target of gaze.

At shorter viewing durations, the social signal (i.e., the eye cue) will be less reliable. This will also be true of the presumed relative visual salience of the various locations in the gazed-upon image, a contextual cue that requires time to be evaluated by the observer. By examining the extent to which image salience influences gaze judgments at different timescales, we attempted to gain insight into the rates at which useful information from different sources is extracted to inform these judgments.

### Methods

#### Subjects

Forty-one undergraduates at Indiana University received course credit for their participation in the experiment.

#### Stimuli

Experiment 2 utilized the same set of 33 photographs of the gazer as in Experiment 1, and projected the same 165 color images into these photographs.

#### Procedure

The experiment consisted of two blocks, each consisting of 165 consecutive trials. Before the first trial of each block, four photographs were displayed in succession, each for 1 s—the same calibration employed in Experiment 1. Each trial began with a black fixation cross, presented at the center of the screen for 1.4 s against a gray background. The subject was then presented with a static scene. These scenes featured each of the 33 photographs of the gazer (fixating on 33 respective locations) five times per block, and these 33 × 5 = 165 total trials were randomly shuffled. One of 165 color images from the stimulus set was randomly assigned to each of these 165 trials and projected into the frame in front of the gazer; thus, the projected image and the direction of the subject's gaze in the photograph were randomly paired and presented simultaneously. The first three trials of each block were considered practice and were excluded from analysis.

Each scene was displayed for one of five different durations: 150, 300, 600, 1200, or 2400 ms. This viewing duration was crossed with the gazer's 33 possible eye directions such that every combination of viewing duration and gaze direction was viewed once per subject per block. After the presentation of a scene, the scene was replaced with a Gaussian noise mask. Only a black frame remained visible to the subject, demarcating the edges of where the projected image had been situated. A 10- × 10-pixel red square appeared at a random location within the frame, and could be controlled by the mouse. The subject indicated with a mouse click where, within the frame, he or she believed that the gazer had been looking in the image (before the scene had been masked). There was no enforced time limit for response; after the subject clicked, the next trial began.

### Results

We examined whether the relative salience of locations in the projected image influenced subjects' gaze judgments differently at different timescales. To preface these analyses, the answer to this question appears to depend on one's working definition of “salience.”

**Figure 5:**
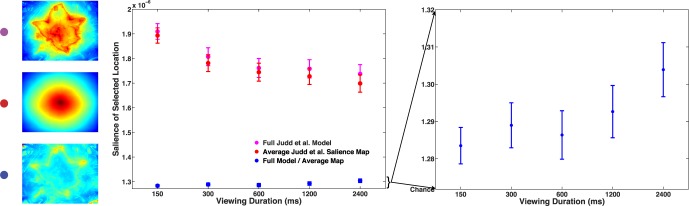
Experiment 2. The average salience of the location selected by subjects, across viewing duration conditions (the *x*-axis is log scaled). Salience computed with the full Judd et al. algorithm is in magenta; salience computed using the average salience map across the entire set of stimulus images is displayed in red; the computed salience of the location, divided by the average salience across all images at that location, is displayed in blue, and zoomed in on in the right panel. Error bars represent ± *SEM*.

Using the full Judd et al. (2009) algorithm—uncorrected for center bias—as our model of salience, we found that when subjects were only allowed to view a scene for a short time, they tended to judge more salient locations as the target of gaze (Figure 5; The mean slope of linear regressions of viewing duration vs. location salience—fit to each individual subject—was significantly negative, *t*[40] = −6.71, *p* < 0.001)—that is, the more limited the subject's exposure to the scene, the more subjects' relied on the prior. However, a more thorough examination of the data reveals that this interpretation of the data does not tell the full story.

As in Experiment 1, we averaged the salience maps corresponding to all of the stimulus images, resulting in a composite map reflecting the global tendency for locations toward the center of each image to be salient. As illustrated in Figure 5, the tendency for subjects to rely more on image salience at shorter viewing durations can be attributed to this center bias; if one only used the composite average map to predict subjects responses, one would observe the same, strongly negative relationship between viewing duration and salience, *t*(40) = −8.68, *p* < 0.001.

On the other hand, if one controlled for the typical salience across images at each location in the scene (by dividing off the average salience map from the full salience map generated for each image), one could instead ask, “How salient was the location selected by the subject, compared to the average salience at that location?”—that is, above what is typical for that spatial location across images. And in this case, the effect reverses direction: Subjects tended to select more salient locations when they had more time to view the scenes, *t*(40) = 2.40, *p* = 0.02.

Thus, the timescale of the effect of image salience on gaze perception depends on one's working definition of “salience.” Further exploring this discovery, we recomputed the salience maps for our set of images using the Judd et al. (2009) algorithm, but this time only included the low-level features of the images in the computation of the maps. We therefore isolated these features from explicit center bias as well as mid- and high-level features (including horizon, car, face, and people detection).^1^

**Figure 6:**
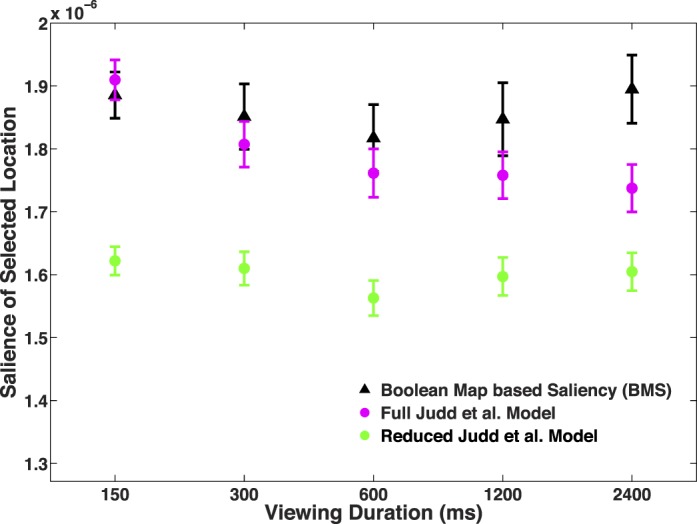
Experiment 2. The average salience of the location selected by subjects, across viewing duration conditions (the *x*-axis is log scaled). Salience is computed three ways: the Boolean Map based–Saliency model (black), the full Judd et al. (2009) algorithm (magenta), and a reduced version of the Judd et al. algorithm that is only computed with respect to low-level features (green). Error bars represent ± *SEM*.

Using this reduced model, we find a different result (Figure 6). With the exception of a possible dip at 600 ms, the locations selected by subjects tend to be approximately equally salient across timescales (the mean slope of the regression of viewing duration vs. salience was not significantly different from zero, *t*[40] = −0.14, *p* = 0.89). Using another salience algorithm (Boolean Map–based Saliency; Zhang & Sclaroff, 2013)—which likewise employs only low-level features, and has been validated on this same set of images—we find a similar pattern of results, *t*(40) = 0.79, *p* = 0.44.

With respect to both the reduced Judd et al. (2009) model and the Boolean Map–based Saliency model, more salient locations still tend to be more centrally located in images, even without the inclusion of any explicit center bias as a feature. Given the large and robust tendency for subjects to have a strong center bias at short viewing durations, our best interpretation of the data conveyed in Figures 5 and 6 is that center bias tends to dominate at shorter viewing durations, but other salient, low-level features of the images may operate at longer timescales. Whereas center bias is present at the start of each trial—truly “prior” to the stimulus—the salient features of the images need to be computed, and therefore may come to influence subject's gaze judgments at later timescales. These countervailing effects likely produce the flat or U-shaped curves observed in Figure 6.

In summary, when subjects were only allowed to view a person gazing at an image briefly, they showed a strong center bias in their estimates of where the gazer was looking in the projected image. Greater reliance on this prior bias was predictable, because the processed signals from both the gazer's eyes and the contextual image salience are less reliable at shorter timescales. As the viewing duration was increased, the influence of salience with respect to the local features of the gazed-upon image became more apparent.

## Discussion and conclusions

In this article, we developed a Bayesian model of gaze perception, which takes into account both cues from the gazer's eyes and prior salience information present in the visual scene. Via a quantitative model comparison, we demonstrated in Experiment 1 that this full Bayesian model accounts for the performance of most subjects better than a reduced model that only takes the eye cues into account. The full Bayesian model also easily outperforms a model that incorporates incorrect (and empirically useless) salience information. We consider these data to be strong preliminary support for a Bayesian account of human gaze perception, and of closely related social processes like gaze following and joint attention.

The data from Experiment 1 may also indicate that a subset of subjects (∼18%) utilized only the cues from the eyes of the gazer. These individual differences in strategy raise many questions to be addressed in future experiments: Was the salience algorithm we employed a poor model for where a minority of subjects expect other people will look in the scene? Is the tendency to use one strategy over the other relatively stable to the individual? Would certain clinical populations (e.g., individuals with autism spectrum disorder) show a systematic tendency to use one strategy versus the other? In other words, were the individual differences we observed meaningful?

We emphasize that we do not mean to present this paper as a study of how gaze perception relates to salience (defined in any one particular way, via any specific algorithm), as a visual feature in itself. Rather, we use computed salience (according to one algorithmic approach) as a simplified stand-in (that is, a model) for the predictive computation of which locations in a scene would be expected to draw another person's visual attention. Most subjects' judgments revealed that they were at least implicitly sensitive to these a priori expectations, which were apparently correlated with the output of the salience model we employed. On the other hand, we acknowledge that the locations in scenes at which one would expect the gazer to direct his attention were likely to also be intrinsically salient to the subject him- or herself. That is, expected salience vis-à-vis the gazer and subjective salience are strongly correlated.

A clever experiment might be able to decouple these two qualities. For example, the experimenter could tell the subject that the gazer is searching for red objects in each scene, and examine whether subjects then tend to judge the redder objects or areas of the scene as being more likely to be the target of gaze. This would manipulate the subject's expectations of what is likely to be salient to the gazer—in one particular context—under the assumption that red objects become no more salient to the subject, per se. We have little doubt that subjects could alter their strategies to modulate their performance in such a task, especially with practice. However, this might be achieved by tapping into higher level processes and decisional criteria that may not be representative of gaze perception as employed more naturally and reflexively in more typical situations. Further, if the subject knew that particular features of a scene were likely to be especially salient to the gazer, it is quite plausible that these features would irresistibly become more salient to the subject him- or herself. This would defeat the purpose an experiment designed to decouple these qualities.

The bad news, therefore, is that what one expects to be salient to another and what is salient to oneself may indeed be hopelessly confounded for the purposes of an empirical study like this. The good news is that in most naturalistic situations, these two qualities are also confounded with one another. If there is something that draws one person's attention, it is likely to draw others' attention as well (Borji, Parks, & Itti, 2014). The human perceptual apparatus does not lament this correlation, but exploits it: One can continually leverage the successful computation of one of these qualities to help infer the other. That these complementary processes (predicting the probable locations of salient objects from another's gaze and inferring the target of another's gaze from the locations of salient objects) provide ample feedback for one another may be the basis for efficient learning during early social development (Triesch, Jasso, & Deák, 2007).

A Bayesian account of eye gaze perception makes several specific predictions for how various experimental manipulations will affect gaze judgments. For example, the noisier the social signal, the more the observer should rely on prior information. In Experiment 2, we manipulated the amount of time subjects' were exposed to scenes. Because at shorter durations, subjects' exposure to both eye cues and image salience cues was limited, the influence of both of these cues was enhanced at longer durations. Spatial biases—which are truly prior to the stimulus—prevailed at earlier timescales. An analogous result with respect to spatial biases in gaze judgments was also observed by Mareschal, Calder, Dadds, and Clifford (2013), who found subjects' prior bias toward direct eye contact was modulated by the amount of noise the experimenters added to the observed eyes.

We expect that many other manipulations like this could also be applied to the basic experimental framework presented in this paper, with analogous results. Besides varying stimulus duration or adding noise to the gazer's eyes (e.g., via blurring), one could manipulate the size or contrast of the stimulus, or the distance between the gazer and the gazed-upon surface in the scene. The perceptual consequences of each of these manipulations could then be interpreted within the context of this Bayesian treatment, providing additional insight into the nature of human gaze perception.

## Supplementary Material



## Footnotes

1 These higher level features likely operate on their own respective timescales in terms of their influence on subjects gaze judgments. However, the higher level aspects of the Judd et al. salience model were difficult to analyze in isolation for numerous reasons: (a) Higher level features (such as faces) also tend to be more centrally located, and more salient with respect to low-level features; (b) The higher level maps consist of discrete bounding boxes, which are often as large or larger than the area of an image that might be plausibly selected by the subject; thus, it is unclear whether they can be used as sensitive measures of performance difference across experimental conditions; and (c) High-level features (faces, people, and cars) were only present in a subset of the images, and the algorithms used to detect these features did not have perfect sensitivity. These considerations limited our ability and statistical power to analyze the timescales of the influence of these higher level features on subject performance.
