# Supplementary figures and images for: Prior expectations about where other people are likely to direct their attention systematically influence gaze perception
Source: J Vis. 2016 Feb 5;16(3):7. doi: 10.1167/16.3.7 (PMC4747336; doi:10.1167/16.3.7)

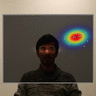

Supplement: Supplementary file 1 [file i1534-7362-16-3-7-icon.gif]
